# Epidemiology of SARS-CoV-2 transmission and superspreading in Salt Lake County, Utah, March–May 2020

**DOI:** 10.1371/journal.pone.0275125

**Published:** 2023-06-23

**Authors:** Joseph Walker, Tiffany Tran, Brooke Lappe, Paul Gastanaduy, Prabasaj Paul, Ian T. Kracalik, Victoria L. Fields, Adriana Lopez, Amy Schwartz, Nathaniel M. Lewis, Jacqueline E. Tate, Hannah L. Kirking, Aron J. Hall, Eric Pevzner, Ha Khong, Maureen Smithee, Jason Lowry, Angela Dunn, Tair Kiphibane, Cuc H. Tran

**Affiliations:** 1 COVID-19 Response Team, U.S. Centers for Disease Control and Prevention, Atlanta, Georgia, United States of America; 2 Epidemic Intelligence Service, U.S. Centers for Disease Control and Prevention, Atlanta, Georgia, United States of America; 3 Salt Lake County Health Department, Salt Lake City, Utah, United States of America; 4 Utah Department of Health, Salt Lake City, Utah, United States of America; Flinders University, AUSTRALIA

## Abstract

**Background:**

Understanding the drivers of SARS-CoV-2 transmission can inform the development of interventions. We evaluated transmission identified by contact tracing investigations between March–May 2020 in Salt Lake County, Utah, to quantify the impact of this intervention and identify risk factors for transmission.

**Methods:**

RT-PCR positive and untested symptomatic contacts were classified as confirmed and probable secondary case-patients, respectively. We compared the number of case-patients and close contacts generated by different groups, and used logistic regression to evaluate factors associated with transmission.

**Results:**

Data were collected on 184 index case-patients and up to six generations of contacts. Of 1,499 close contacts, 374 (25%) were classified as secondary case-patients. Decreased transmission odds were observed for contacts aged <18 years (OR = 0.55 [95% CI: 0.38–0.79]), versus 18–44 years, and for workplace (OR = 0.36 [95% CI: 0.23–0.55]) and social (OR = 0.44 [95% CI: 0.28–0.66]) contacts, versus household contacts. Higher transmission odds were observed for case-patient’s spouses than other household contacts (OR = 2.25 [95% CI: 1.52–3.35]). Compared to index case-patients identified in the community, secondary case-patients identified through contract-tracing generated significantly fewer close contacts and secondary case-patients of their own. Transmission was heterogeneous, with 41% of index case-patients generating 81% of directly-linked secondary case-patients.

**Conclusions:**

Given sufficient resources and complementary public health measures, contact tracing can contain known chains of SARS-CoV-2 transmission. Transmission is associated with age and exposure setting, and can be highly variable, with a few infections generating a disproportionately high share of onward transmission.

## Introduction

Current understanding of Severe acute respiratory syndrome coronavirus 2 (SARS-CoV-2) epidemiology highlights a high degree of variability in the transmission patterns across and within communities [1–3], reflecting context- and exposure setting-specific factors, as well as differences in the efficacy of the control strategies that have been implemented. A better understanding of determinants of SARS-CoV-2 transmission in various settings and the impact of mitigation in curbing transmission is key to inform local prevention and public health response recommendations.

Early in the pandemic, the Salt Lake County Health Department (SLCoHD) conducted contact tracing of laboratory-confirmed SARS-CoV-2 cases among residents in Salt Lake County, Utah, to reduce transmission through isolation and quarantine. As part of their efforts, demographic and clinical data were collected from cases and their close contacts, providing an opportunity to understand contact tracing and transmission patterns. In a previous study, we leveraged these data to describe contact tracing and estimate the resources needed to run an effective contact tracing program [4]. In this study, we use these data to characterize the risk factors and heterogeneity associated with SARS-CoV-2 transmission in various settings and to quantify the impact of contact tracing on SARS-CoV-2 transmission.

## Methods

### Contact tracing context

SLCoHD routinely investigates and traces contacts of coronavirus disease 2019 (COVID-19) case-patients in Salt Lake County, Utah. For this study, we define an index case-patient as the first confirmed case-patient of the transmission chain which triggered contact tracing. These case-patients were identified from the community and reported to SLCoHD after a positive SARS-CoV-2 test result. Secondary case-patients were defined as confirmed or probable case-patients identified through contact tracing; confirmed case-patients received a positive SARS-CoV-2 real-time reverse transcription-polymerase chain reaction (RT-PCR) test result, regardless of symptoms, and probable case-patients were close contacts who developed symptoms but were not tested. We used the broader term case-patients to indicate both index case-patients and secondary case-patients collectively.

Due to time and resource constraints, it was not possible to abstract data from all confirmed case-patients reported during our study period (March 12 –May 3). In order to derive a representative sample of index case-patients and their associated chains of traced contacts, we attempted to systematically select every 10^th^ confirmed case-patient from a line-list (however, this interval had to be increased periodically over the course of the study period, so that data abstraction could keep pace with the rapidly growing case volume). After excluding case-patients who lacked sufficient data or were traced from a previously identified case-patient, we reviewed contact tracing data to collect information on the demographics, symptoms, comorbidities, clinical outcomes (e.g., hospitalization, death), laboratory results, and exposure settings, as applicable, of the confirmed case-patients and up to six generations of their traced contacts. In line with CDC guidance [5], close contacts were defined as persons who spent at least 15 minutes within 6 feet of a confirmed or probable case-patient in the period starting 2 days before the case-patient’s onset of symptoms or, if the case-patient was asymptomatic, 2 days before their sample collection date, until the case-patient was identified and isolated. Contacts were routinely monitored, either by a daily phone call or text message, for presence of symptoms for 14 days after their last exposure to a confirmed or probable case-patient. In addition to the index case-patients, contact tracing was performed for any contact that tested positive or became symptomatic during the monitoring period (i.e., all confirmed and probable secondary case-patients, as well as symptomatic contacts that went on to test negative, and therefore were not classified as secondary case-patients). This process was repeated until no further positive or symptomatic contacts could be identified.

To quantify the impact of contact tracing on SARS-CoV-2 transmission, we compared the mean number of secondary case-patients and close contacts generated by two types of confirmed case-patients: 1) index case-patients, who were reported to SLCoHD after receiving a positive test in the community (“identified via community testing”), and 2) confirmed secondary case-patients, who were identified by SLCoHD during contact tracing investigations and subsequently received a positive test (“identified via contact tracing”) (Table 1). 95% confidence intervals (CI) for the mean number of secondary case-patients and contacts generated by each case-patient, and *P* values for the difference in means between groups, were obtained by applying a bias-corrected and accelerated (BCa) bootstrapping procedure with 10,000 replicates to the data, using the *boot* R package (this form of bootstrapping automatically transforms the data to normalize it and stabilize its variance, thereby producing more accurate confidence intervals in small-sample situations). We also compared the secondary attack rate and test percent-positivity between contacts of index and confirmed secondary case-patients, using Z-tests with Yate’s continuity correction to calculate confidence intervals and *P* values for differences in proportions.

**Table 1:** Mean secondary case-patients^a^ and close contacts per case-patient, and secondary attack rates and test positivity rates among contacts, by mode of detection.

	Mode of Detection	
	Community Testing (Referent)	Contact Tracing
No. of Confirmed Case-Patients	184	285
No. of Traced Contacts of Confirmed Case-Patients	922	485
Mean Secondary Case-Patients^a^ per Confirmed Case-Patient	1.55 (1.32–1.80)	0.28** (0.21–0.38)
Confirmed	1.21 (1.02–1.43)	0.22** (0.16–0.29)
Household	1.10 (0.91–1.32)	0.20** (0.14–0.28)
Confirmed	0.85 (0.70–1.05)	0.16** (0.11–0.22)
Non-household	0.45 (0.34–0.59)	0.07** (0.04–0.11)
Confirmed	0.36 (0.27–0.47)	0.06** (0.03–0.09)
Mean Traced Contacts per Confirmed Case-Patient	5.01 (4.46–5.70)	1.70** (1.33–2.35)
Household	2.97 (2.67–3.31)	0.80** (0.62–1.01)
Non-household	2.04 (1.55–2.70)	0.90** (0.60–1.50)
Tested	2.47 (2.14–2.85)	0.62** (0.48–0.78)
Household	1.65 (1.41–1.96)	0.33** (0.25–0.43)
Non-household	0.82 (0.63–1.08)	0.29** (0.20–0.41)
Secondary Attack Rate^b^	31% (28%–34%)	16%** (13%–20%)
Household	37% (33%–31%)	25%** (20%–32%)
Non-household	22% (18%–27%)	8%** (5%–12%)
Positivity Among Tested Contacts	49% (44%–54%)	35%** (28%–43%)
Household	52% (46%–57%)	47% (37%–58%)
Non-household	44% (36%–52%)	21%** (13%–31%)

Note: 95% confidence interval presented in parentheses.

*P<0.05

**P<0.01

^a^Includes both confirmed and probable secondary case-patients of only confirmed case-patients.

^b^Secondary attack rates among contacts were calculated as the proportion of contacts that were confirmed (PCR positive for SARS-CoV-2) or probable (symptomatic but untested) secondary case-patients among all contacts.

Finally, to quantify differences in transmission potential by age, we performed the same comparisons (mean number of secondary case-patients and close contacts generated, and secondary attack rates and test-percent positivity among associated contacts) between different age-groups of confirmed case-patients (Table 2).

**Table 2:** Mean secondary case-patients^a^ and close contacts per case-patient, and secondary attack rates and test positivity rates among contacts, by age of case-patient.

	Age of Confirmed Case-Patient (years)			
	<18	18–44 (Referent)	45–64	≥65
No. of Confirmed Case-Patients	40	249	138	41
No. of Traced Contacts of Confirmed Case-Patients	11	885	431	80
Mean Secondary Case-Patients^a^ per Confirmed Case-Patient	0.05** (0.00–0.12)	0.90 (0.74–1.10)	0.82 (0.62–1.07)	0.61 (0.32–1.20)
Confirmed	0.05** (0.00–0.12)	0.67 (0.54–0.83)	0.68 (0.50–0.91)	0.54 (0.27–1.02)
Household	0.05** (0.00–0.12)	0.65 (0.52–0.85)	0.57 (0.41–0.75)	0.46 (0.22–0.93)
Confirmed	0.05** (0.00–0.12)	0.47 (0.37–0.61)	0.49 (0.34–0.67)	0.39 (0.17–0.78)
Non-household	0.00**	0.26 (0.18–0.36)	0.25 (0.16–0.36)	0.15 (0.05–0.32)
Confirmed	0.00**	0.20 (0.14–0.28)	0.20 (0.12–0.30)	0.15 (0.05–0.32)
Mean Traced Contacts per Confirmed Case-Patient	0.28** (0.08–0.62)	3.55 (3.05–4.16)	3.12 (2.44–4.33)	1.95* (1.27–3.00)
Household	0.22** (0.05–0.62)	2.00 (1.72–2.30)	1.52* (1.23–1.86)	1.41 (0.88–2.37)
Non-household	0.05** (0.00–0.12)	1.56 (1.18–2.09)	1.60 (1.03–2.76)	0.54** (0.22–1.10)
Tested	0.20** (0.05–0.50)	1.49 (1.25–1.78)	1.54 (1.22–1.93)	1.00* (0.61–1.56)
Household	0.20** (0.05–0.50)	0.92 (0.74–1.15)	0.96 (0.74–1.22)	0.76 (0.41–1.27)
Non-household	0.00**	0.57 (0.43–0.74)	0.59 (0.41–0.89)	0.24* (0.10–0.41)
Secondary Attack Rate^b^	18% (3%–52%)	25% (23%–28%)	26% (22%–31%)	31% (22%–43%)
Household	22% (4%–60%)	32% (28%–37%)	38% (31%–45%)	33% (21%–46%)
Non-household	0% (0%–80%)	16% (13%–21%)	15% (11%–21%)	27% (12%–50%)
Positivity Among Tested Contacts	25% (4%–64%)	45% (40%–50%)	44% (37%–51%)	54% (38%–69%)
Household	25% (4%–64%)	51% (45%–58%)	51% (42%–60%)	52% (33%–69%)
Non-household	NA	35% (28% - 44%)	33% (23% - 45%)	60% (27% - 86%)

Note: 95% confidence interval presented in parentheses.

*P<0.05

**P<0.01

^a^Includes both confirmed and probable secondary case-patients of only confirmed case-patients.

^b^Secondary attack rates among contacts were calculated as the proportion of contacts that were confirmed (PCR positive for SARS-CoV-2) or probable (symptomatic but untested) secondary case-patients.

### Infectiousness and susceptibility

To investigate factors associated with infectivity of SARS-CoV-2, we calculated secondary attack rates among contacts based on case-patients’ characteristics (age, sex, race/ethnicity), presence of cough, hospitalization, and clinical outcome (recovered or died). To investigate factors associated with susceptibility to SARS-CoV-2, we calculated secondary attack rates among contacts based on close contacts’ characteristics. Finally, we compared secondary attack rates among contacts based on the type of exposure setting (i.e., household, social, workplace, healthcare, and other). Generalized linear models were fit to estimate the odds ratio of becoming a secondary case (either confirmed or probable) for each infectivity, susceptibility, and setting factor. To account for possible confounding effects between variables, we developed multivariable models, including all variables with univariate *P* values <0.2. Contacts of probable secondary case-patients were excluded from both the univariate and multivariate analyses. These analyses were first conducted across all exposure settings and repeated separately for household and non-household contacts.

All data were analyzed using R and STATA 16.1 statistical software programs.

### Ethical considerations

This activity was reviewed and approved by the CDC Human Subject Review Office and was conducted consistent with applicable federal law and CDC policy. See e.g., 45 C.F.R. part 46, 21 C.F.R. part 56; 42 U.S.C. §241(d); 5 U.S.C. §552a; 44 U.S.C. §3501 et seq.

The requirement for participant consent was waived by the CDC Human Subject Review Office as this activity was a secondary analysis of data collected as part of a public health response. However, given that this data describes the transmission dynamics and contact networks of up to six generations of traced contacts, the data contains potentially identifying information as determined by the CDC Human Subject Review Office. Data requests can be sent to the CDC Human Subject Review Office through the CDC Office of Science (OADS@cdc.gov) using the STARS ID 0900f3eb81ce58e5.

## Results

### Impact of contact tracing on secondary case-patients

We used contact tracing data collected between March 12–May 3, 2020, during which a line-list of 2,757 confirmed case-patients was identified. Systematic sampling from this line-list produced an initial selection of 229 confirmed case-patients, of which 45 were excluded (n = 12 were contacts of an earlier case-patient, and n = 33 had insufficient data), resulting in a final sample of 184 index case-patients and 1,499 close contacts for analysis. Index case-patients (identified through community testing) were linked to a mean of 5.01 (95% CI: 4.46–5.70) contacts and 1.55 (95% CI: 1.32–1.80) secondary case-patients (31% secondary attack rate). Lab-confirmed secondary case-patients (identified through contact tracing) generated a mean of 1.70 (95% CI: 1.33–2.35) contacts and 0.28 (95% CI: 0.21–0.38) secondary case-patients (16% secondary attack rate); differences in all 3 measures were statistically significant at *P*<0.001 (Table 1).

Differences remained statistically significant when the analysis was limited to household contacts and non-household contacts. Contacts of secondary case-patients identified also had significantly lower test percent-positivity than contacts of index case-patients, both overall (35% vs. 49%, *P*<0.001), and for non-household contacts (21% vs. 44%, *P*<0.001), but not among household contacts (47% vs. 52%). Case-patients aged 18–44 years generated the greatest mean number of secondary case-patients and contacts (0.90 and 3.55 per case-patient, respectively) (Table 2). In comparison, confirmed case-patients aged <18 years generated significantly fewer secondary case-patients (0.05 per case-patient, *P<0*.*001*), and case-patients aged <18 and ≥65 years generated significantly fewer contacts (0.28 and 1.95 per case-patient, *P<0*.*001* and *P = 0*.*002*, respectively). Secondary attack rates and test percent-positivity among contacts did not vary significantly between age-groups of the case-patients.

### Demographic characteristics of case-patients and their close contacts

The median ages of index case-patients and close contacts were 38 years (range = 15–93 years; interquartile range [IQR] = 28–53 years) and 29 years (range = 1–87 years; IQR = 18–45 years), respectively (Table 3). Compared to index case-patients, a greater proportion of probable secondary case-patients were aged <18 years. Non-Hispanic/Latino White and Hispanic/Latino were the most common racial/ethnic groups among both index case-patients (47%, 36%) and close contacts (29%, 34%).

**Table 3:** Demographic and clinical characteristics of index case-patients^a^ and their contacts.

Characteristic	Index Case-Patients^a^	Contacts	Secondary Confirmed Case-Patients	Secondary Probable Case-Patients	Non-case Contacts
	n = 184	n = 1,499	n = 285	n = 89	n = 1,125
Age, years, median (IQR)	38 (28–53)	29 (18–45)	36 (24–52)	20 (10.5–30.5)	28.5 (17–44)
Age group, years					
<18	3 (2%)	349 (23%)	37 (13%)	38 (43%)	274 (24%)
18–44	102 (55%)	702 (47%)	147 (52%)	37 (42%)	518 (46%)
45–64	61 (33%)	292 (19%)	77 (27%)	9 (10%)	206 (18%)
≥65	18 (10%)	74 (5%)	23 (8%)	3 (3%)	48 (4%)
Unknown	0 (0%)	82 (5%)	1 (<1%)	2 (2%)	79 (7%)
Sex					
Male	91 (49%)	711 (47%)	139 (49%)	43 (48%)	529 (47%)
Female	93 (51%)	729 (49%)	145 (51%)	45 (51%)	539 (48%)
Unknown	0 (0%)	59 (4%)	1 (<1%)	1 (1%)	57 (5%)
Race/Ethnicity					
Hispanic	66 (36%)	509 (34%)	124 (44%)	35 (39%)	350 (31%)
White, non-Hispanic	86 (47%)	438 (29%)	99 (35%)	28 (31%)	311 (28%)
Black or African American, non-Hispanic	4 (2%)	22 (1%)	5 (2%)	0 (0%)	17 (2%)
Asian, non-Hispanic	7 (4%)	64 (4%)	25 (9%)	4 (4%)	35 (3%)
Other^b^, non-Hispanic	2 (1%)	60 (4%)	14 (5%)	6 (7%)	40 (4%)
Unknown^c^	19 (10%)	406 (27%)	18 (6%)	16 (18%)	372 (33%)
Exposure setting	–				
Household		821 (55%)	202 (71%)	65 (73%)	554 (49%)
Non-household^d^		678 (45%)	83 (29%)	24 (27%)	571 (51%)
Healthcare		38 (3%, 6%)	2 (1%, 2%)	0 (0%, 0%)	36 (3%, 6%)
Workplace		308 (21%, 45%)	36 (13%, 43%)	7 (8%, 29%)	265 (24%, 46%)
Social		229 (15%, 34%)	34 (12%, 41%)	14 (16%, 58%)	181 (16%, 32%)
Other^e^		52 (3%, 8%)	6 (2%, 7%)	2 (2%, 8%)	44 (4%, 8%)
Unknown		51 (3%, 8%)	5 (2%, 6%)	1 (1%, 0.4%)	45 (4%, 8%)

^a^In this table, index-case patients refer to confirmed case-patients identified in the community and reported to SLCoHD, excluding confirmed or probable secondary case-patients identified through contact tracing.

^b^Other race/ethnicity includes American Indian/Alaska Native, non-Hispanic; Native Hawaiian/Other Pacific Islander, non-Hispanic; and Two or More Races/Other, non-Hispanic.

^c^Unknown race/ethnicity includes individuals of unknown race who are not known to be Hispanic and individuals of unknown ethnicity.

^d^For individual non-household exposure settings, the first and second numbers within the parenthesis indicate the proportion the individual setting represents of all exposure settings, and of non-household settings, respectively.

^e^Other non-household settings include school and daycare, conference, family members not living in the household, retail stores.

Almost all case-patients reported at least one symptom and 30% of index case-patients reported at least one underlying health condition. Of the 469 confirmed case-patients in this analysis, 40 were hospitalized (8.5%) and 7 died (1.5%). Among 1,125 non-case contacts, 162 (14%) presented symptoms but tested negative for SARS-CoV-2. Additional data on the clinical presentation, comorbidities, and outcomes of case-patients are available in Supporting information (S1 and S2 Tables).

Households were the most common setting of exposure (55%), while non-household contacts were most commonly exposed in workplace (45%) and social settings (34%) (Table 3).

### Characteristics associated with infectiousness and susceptibility

The effect of individual- and setting-level characteristics on per-exposure infectiousness and susceptibility across both household and non-household settings are shown in Table 4. With respect to infectivity, attack rates among contacts were significantly higher when the case-patient was Hispanic/Latino or non-White compared to non-Hispanic/Latino White (32% vs. 26%, respectively; univariate OR = 1.36, *P* = 0.02), although the race/ethnicity of the case-patient did not remain significant after controlling for the setting of exposure, the sex of the case-patient, and the age and race/ethnicity of the contact (multivariate OR = 0.93, *P* = 0.71). With respect to susceptibility, attack rates were lower among contacts aged <18 years, compared to those aged 18–44 years (21% vs. 27%, respectively; univariate OR = 0.74, *P* = 0.06). Attack rates were significantly lower in each of the non-household settings (workplace, healthcare, social gatherings, other) compared to household settings. In the multivariate model, contacts aged <18 years and exposures in social and workplace settings, rather than in the household, remained independently associated with lower attack rates among contacts.

**Table 4:** Secondary attack rates among 1407 close contacts based on case-patient^a^ characteristics, their own characteristics, and exposure setting.

			Univariate			Multivariate	
Case-Patient Characteristic		No. of Contacts	No. of Secondary Case-Patients (Secondary Attack Rate)^b^	OR (95% CI)	*P*	OR (95% CI)	*P*
Age (years)	<18	11	2 (18%)	0.65 (0.10–2.55)	0.59		
	18–44	885	225 (25%)	REFERENT			
	45–64	431	113 (26%)	1.04 (0.80–1.35)	0.76		
	≥65	80	25 (31%)	1.33 (0.80–2.17)	0.26		
Sex	Female	716	172 (24%)	REFERENT			
	Male	691	193 (28%)	1.23 (0.97–1.56)	0.09^f^	0.97 (0.73–1.29)	0.84
Race/Ethnicity^c^^,^^d^	Non-Hispanic White	563	147 (26%)	REFERENT			
	Hispanic or Non-White	586	190 (32%)	1.36 (1.05–1.75)	0.02^f^	0.93 (0.61–1.40)	0.71
Cough^d^	Yes	1079	283 (26%)	REFERENT			
	No	301	80 (27%)	1.02 (0.76–1.36)	0.90		
Hospitalization^d^	No	1270	334 (26%)	REFERENT			
	Yes	132	30 (23%)	0.82 (0.53–1.25)	0.37		
Outcome^d^	Died	7	3 (43%)	2.06 (0.40–9.39)	0.35		
	Recovered	1334	356 (27%)	REFERENT			
Close Contact Characteristics							
Age (years)^d^	<18	326	70 (21%)	0.74 (0.54–1.01)	0.06^f^	0.55 (0.38–0.79)	<0.01
	18–44	668	180 (27%)	REFERENT			
	45–64	281	86 (30%)	1.20 (0.88–1.62)	0.25	1.37 (0.95–1.96)	0.09
	≥65	73	26 (36%)	1.50 (0.89–2.48)	0.12^f^	1.61 (0.85–3.02)	0.14
Sex^d^	Female	685	185 (27%)	REFERENT			
	Male	675	178 (26%)	0.97 (0.76–1.23)	0.79		
Race/Ethnicity^d^	Non-Hispanic White	421	125 (30%)	REFERENT			
	Hispanic or Non-White	631	216 (34%)	1.23 (0.95–1.61)	0.12^f^	1.30 (0.85–1.98)	0.22
Exposure Setting^d^							
Type	Household	774	261 (34%)	REFERENT			
	Healthcare	38	2 (5%)	0.11 (0.02–0.36)	<0.01^f^	0.22 (0.01–1.41)	0.17
	Work	299	43 (14%)	0.33 (0.23–0.47)	<0.001^f^	0.36 (0.23–0.55)	<0.001
	Social	212	48 (23%)	0.58 (0.40–0.81)	<0.01^f^	0.44 (0.28–0.66)	<0.001
	Other^e^	45	6 (13%)	0.30 (0.11–0.67)	<0.01^f^	0.39 (0.11–1.07)	0.10

^a^In this table, case-patients refer to laboratory confirmed case-patients identified in the community and reported to SLCoHD, as well as laboratory confirmed secondary case-patients identified through contact tracing who had their close contacts traced; probable (symptomatic but untested) secondary case-patients were excluded as case-patients from these analyses.

^b^Secondary attack rates among contacts were calculated as the proportion of contacts that were confirmed (tested positive SARS-CoV-2) or probable (symptomatic but untested) secondary case-patients.

^c^Hispanic or Non-White includes Hispanic; Black or African American, non-Hispanic; Asian, non-Hispanic; American Indian/Alaska Native, non-Hispanic; Native Hawaiian/Other Pacific Islander, non-Hispanic; or Two or More Races/Other, non-Hispanic.

^d^Data were missing for the following variables: race/ethnicity (case-patients of 258 contacts and 355 contacts), cough (case-patients of 27 contacts), hospitalization (case-patients of 5 contacts), outcome (case-patients of 66 contacts), age (59 contacts), sex (47 contacts), exposure setting (39 contacts).

^e^Other non-household exposure settings include school and daycare, conference, family members not living in the household, retail stores.

^f^Variables with *P* values <0.20 in the univariable analyses were included in the multivariate model. The multivariate model included case-patient sex, case-patient race/ethnicity, contact age, contact race/ethnicity, and exposure setting. Due to concerns for collinearity when including both case-patient and contact race/ethnicity, the analyses were repeated excluding each of these terms in the multivariable model; results were similar with only young age of contact and non-household settings remaining associated with lower secondary attack rates (in these models, all non-household settings, i.e., social, work, healthcare, and other, had significantly lower secondary attack rates compared to household settings).

When the analysis was restricted to household exposures (S1 Table), contacts aged <18 years experienced lower attack rates than those aged 18–44 years, while contacts aged ≥45 years of age or older (i.e., age groups 45–64 and ≥65) or a spouse of the case-patient experienced higher attack rates. These differences were statistically significant in both univariate and multivariate models. In non-household settings (S2 Table), attack rates were significantly elevated among contacts who were Hispanic/Latino or non-white (28% vs. 17% for non-Hispanic/Latino White contacts) or exposed to a case-patient aged ≥65 years (27% vs. 16% of those exposed to a case-patient aged 18–44 years). Secondary attack rates were higher in social settings (23%) compared to workplace (14%) and healthcare (5%) settings, although these differences were not statistically significant when adjusting for other factors.

### Transmission heterogeneity and superspreading

**Fig 1:**
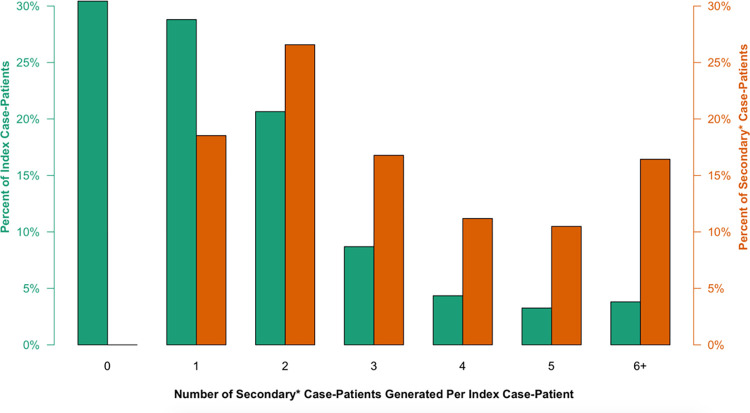
Secondary* case-patients generated by index case-patients. Each pair of bars corresponds to the group of index case-patients who each generated *X* confirmed or probable secondary case-patients (0 to 6+). Green bars indicate the relative size of each group, as a percent of the total number of index case-patients (n = 184), while the orange bars represent the total percent of secondary case-patients directly generated by each group (n = 286). A plurality of index case-patients (30%, 56/184) were not linked to any secondary case-patients at all, while the small group of index-case patients that generated at least 6 secondary case-patients each (4%, 7/184) produced 16% (47/184) of secondary case-patients in the first contact-tracing generation, a highly disproportionate contribution. More broadly, 81% (233/286) of first-generation secondary case-patients were linked to just 41% (75/184) of index case-patient, demonstrating the unevenness of SARS-CoV-2 transmission and important role of superspreading in this population. *Includes secondary case-patients (confirmed and probable) in the first generation of contact tracing (i.e., individuals directly exposed to one of the 184 index case-patients).

The magnitude of onward transmission varied among index case-patients (Fig 1). Most index case-patients (59%, 109/184) generated less than 2 secondary case-patients, and a plurality (30%, 56/184) were not linked to any secondary case-patients. Meanwhile, a small number of index case-patients generated relatively high numbers of secondary case-patients, contributing disproportionately to transmission. We refer to this phenomenon as “superspreading” for the purpose of this report. 4% (7/184) of index case-patients generated 6 or more secondary case-patients, collectively accounting for 16% (47/286) of secondary case-patients in the first contact-tracing generation. More broadly, 81% (233/286) of secondary case-patients in the first contact-tracing generation were generated by only 41% (75/184) of index case-patients (specifically, those that generated 2 or more secondary case-patients) (Fig 1).

## Discussion

With a mean delay of less than a week from infection to onward transmission of SARS-CoV-2, speed and efficiency are prerequisites for effective contact tracing [6, 7]. In the spring of 2020, contact tracing by the SLCoHD facilitated the early identification and management of known close contacts, Fields et al. determined case-patients identified through contact tracing entered quarantine/isolation 5 days earlier than index case-patients on average [4]. Consequently, case-patients who were identified through contact tracing had fewer close contacts, and these contacts had lower secondary attack rates (possibly due to reductions in the cumulative duration and intensity of persistent contacts), with the overall effect being a significant reduction in observed onward transmission. It should be noted that society-wide behavioral changes and community mitigation measures were in place for most of the study period, which likely facilitated more timely and complete contact tracing by reducing the volume of cases and contacts requiring management with finite resources.

Consistent with other studies, we found that a subset of case-patients were responsible for a disproportionate share of exposures and onward transmission events [2, 3, 8–12]. In addition, our observation that confirmed case-patients aged 18–44 years generated more close contacts and secondary case-patients than members of other age-groups underscore the important role of younger adults in SARS-CoV-2 transmission at the population-level [13–15].

We found high transmissibility of SARS-CoV-2 in several settings. The secondary attack rate in household settings of 25%, and in non-household settings of 8%, were higher than the pooled estimates and at the higher end of the ranges reported in a recent systematic review and meta-analysis, 16% (range, 0–45%), and 5% (range, 0–19%), respectively [16]. The heterogeneity in attack rates reported across studies is likely due to differences in the populations evaluated, local disease burden, and study design, including close contact definitions and testing strategies. In the context of communities spending more time at home, and recommendations for isolation and quarantining at home, the higher transmissibility seen in households emphasizes the importance of observing preventive SARS-CoV-2 infection measures, e.g., mask-wearing and social distancing when in public, at work, or attending social events, and getting vaccinated when eligible, to avoid introducing SARS-CoV-2 into the home.

Our contact tracing data allowed us to investigate factors associated with SARS-CoV-2 infectiousness and susceptibility. As seen in prior evaluations of SARS-CoV-2 transmission within households [16], we found that a higher fraction of adult contacts became secondary case-patients compared to children and adolescents. However, these differences may reflect incomplete ascertainment of infection among younger age-groups due to milder or asymptomatic infections. Similar to prior studies [16], spouses of index case-patients in household settings were at higher risk for acquiring SARS-CoV-2, suggesting differential transmission risk from longer and more intimate contact even within households. In non-household settings, attack rates were higher among Hispanic/Latino or non-White contacts compared to non-Hispanic/Latino White contacts; higher secondary attack rates in the workplace setting among these populations (26% vs. 18%, respectively) seem to be the primary driver of this difference.

In sampling for this evaluation, our objective was to select a representative sample of index case-patients and their associated chains of traced contacts to analyze, since time and resource constraints did not allow for detailed data to be abstracted for all case-patients. Sampling was complicated by the fact that many cases had already been reported to SLCoHD by April, when this project commenced. As a result, our sampling method had to accommodate both retrospective selection of cases reported before abstraction began, as well as prospective, real-time selection of cases for the remainder of study period, without knowledge of the total number of cases which would accrue. Given these constraints, simple random sampling was not a practical option for our study, which may make independent replication of our results challenging. However, the form of systematic sampling (not to be confused with convenience sampling) we employed, known as interval sampling (select every *n*^th^ case reported), minimizes bias when simple random sampling is impractical and is commonly used in public health surveillance programs for influenza [17].

Several limitations should be considered. The attack rate may be overestimated because we assumed that case-patients were the only possible source of infection for their associated close contacts [18]. It is possible that the direction of transmission could have been misidentified in some instances, with some traced contacts being the source of infection for the initial case-patient (e.g., overlapping infectious periods), biasing estimates of factors associated with susceptibility and transmissibility. Additionally, inconsistent testing of contacts may have led to underestimation of secondary attack rates, particularly among younger age-groups with a preponderance to mild or asymptomatic infections. Early in the study period, state guidelines prioritized testing of symptomatic close contacts of confirmed case-patients. Later, testing was available for anyone who wanted testing and was approved by their health care provider. Our estimate of a 34% household secondary attack rate is lower than that in studies with universal testing of contacts, e.g., 53% (95% CI: 46–60%) [19]. A substantial proportion (about 25%) of race/ethnicity data was missing for contacts, and we did not collect data on mask usage, social distancing, and other measures that affect transmission levels in different settings. Finally, these data derive from a systematic sample of contact tracing data in one U.S. county, and may not be generalizable to other communities.

During the initial spring 2020 wave of the COVID-19 pandemic, contact tracing was effective at containing known chains of SARS-CoV-2 transmission in Salt Lake County, Utah. Analysis of data collected during these investigations provides insight into multiple aspects of SARS-CoV-2 transmission, including risk factors for infectivity and susceptibility, impact of case-based and population-level control measures, and heterogeneity in onward transmission. We found that transmission risk is associated with age and exposure setting, and a small fraction of infections contributed disproportionately to community transmission. With sufficient resources and complementary public health measures, contact tracing can contain known chains of SARS-CoV-2 transmission.

## Supporting information

S1 Checklist STROBE statement—checklist of items that should be included in reports of observational studies. (DOCX)

S1 Table Secondary attack rates among 774 close contacts in household settings based on case-patient^a^ characteristics and their own characteristics. (DOCX)

S2 Table Secondary attack rates among 633 contacts in non-household settings based on case-patient^a^ characteristics, their own characteristics, and setting of transmission. (DOCX)

S3 Table Number of secondary case-patients generated by 184 index case-patients based on case-patient characteristics. (DOCX)
