# Supplementary material for: Epidemiology of SARS-CoV-2 transmission and superspreading in Salt Lake County, Utah, March–May 2020
Source: PLoS One. 2023 Jun 23;18(6):e0275125. doi: 10.1371/journal.pone.0275125 (PMC10289415; doi:10.1371/journal.pone.0275125)
Supplement: S1 Table — (DOCX) [file pone.0275125.s002.docx]

**S1 Table. Secondary attack rates among 774 close contacts in household settings based on case-patient^a^ characteristics and their own characteristics.**

|  | | | Univariate | | | Multivariate | |
| --- | --- | --- | --- | --- | --- | --- | --- |
| Case-Patient Characteristic |  | No. of Contacts | No. of Secondary Case-Patients (Secondary Attack Rate)^b^ | OR (95% CI) | *P* | OR (95% CI) | *P* |
| Age (years) | <18 | 9 | 2 (22%) | 0.60 (0.09–2.50) | 0.52 | 0.21 (0.01–1.20) | 0.14 |
|  | 18-44 | 497 | 161 (32%) | REFERENT |  |  |  |
|  | 45-64 | 210 | 79 (38%) | 1.26 (0.90–1.76) | 0.18^e^ | 1.07 (0.73–1.56) | 0.73 |
|  | ≥65 | 58 | 19 (33%) | 1.02 (0.56–1.79) | 0.96 | 0.76 (0.38–1.47) | 0.43 |
| Sex | Female | 374 | 123 (33%) | REFERENT |  |  |  |
|  | Male | 400 | 138 (35%) | 1.07 (0.80–1.45) | 0.64 |  |  |
| Race/Ethnicity^c,d^ | Non-Hispanic White | 279 | 100 (36%) | REFERENT |  |  |  |
|  | Hispanic or Non-White | 399 | 151 (38%) | 1.09 (0.79–1.50) | 0.60 |  |  |
| Cough^d^ | Yes | 569 | 204 (36%) | REFERENT |  |  |  |
|  | No | 191 | 57 (30%) | 0.76 (0.53–1.08) | 0.13^e^ | 0.79 (0.52–1.18) | 0.25 |
| Hospitalization^d^ | No | 705 | 242 (34%) | REFERENT |  |  |  |
|  | Yes | 65 | 18 (27%) | 0.73 (0.41–1.27) | 0.28 |  |  |
| Outcome^d^ | Died | 3 | 2 (67%) | 3.79 (0.36–81.7) | 0.28 |  |  |
|  | Recovered | 738 | 255 (35%) | REFERENT |  |  |  |
| Contact characteristics |  |  |  |  |  |  |  |
| Age (years)^d^ | <18 | 273 | 63 (23%) | 0.54 (0.38–0.78) | <0.01^e^ | 0.62 (0.41–0.92) | 0.02 |
|  | 18-44 | 309 | 110 (36%) | REFERENT |  |  |  |
|  | 45-64 | 134 | 63 (47%) | 1.61 (1.06–2.42) | 0.02^e^ | 1.48 (0.93–2.34) | 0.10 |
|  | ≥65 | 39 | 22 (56%) | 2.34 (1.20–4.65) | 0.01^e^ | 2.07 (1.01–4.33) | 0.049 |
| Sex^d^ | Female | 379 | 137 (36%) | REFERENT |  |  |  |
|  | Male | 373 | 122 (33%) | 0.86 (0.63–1.16) | 0.32 |  |  |
| Race/Ethnicity^d^ | Non-Hispanic White | 211 | 90 (43%) | REFERENT |  |  |  |
|  | Hispanic or Non-White | 423 | 157 (37%) | 0.79 (0.57–1.11) | 0.18^e^ | 0.80 (0.56–1.14) | 0.80 |
| Spouse of Case-Patient | Yes | 120 | 60 (50%) | 2.25 (1.52–3.35) | <0.001^e^ | 1.75 (1.08–2.83) | 0.02 |
|  | No | 654 | 201 (31%) | REFERENT |  |  |  |

^a^In this table, case-patients refer to laboratory confirmed case-patients identified in the community and reported to SLCoHD, as well as laboratory confirmed secondary case-patients identified through contact tracing who had their close contacts traced; probable (symptomatic but untested) secondary case-patients were excluded as case-patients from this analyses.

^b^Secondary attack rates among contacts were calculated as the proportion of contacts that were confirmed (tested positive SARS-CoV-2) or probable (symptomatic but untested) secondary case-patients.

^c^Hispanic or Non-White includes Hispanic; Black or African American, non-Hispanic; Asian, non-Hispanic; American Indian/Alaska Native, non-Hispanic; Native Hawaiian/Other Pacific Islander, non-Hispanic; or Two or More Races/Other, non-Hispanic.

^d^Data were missing for the following variables: age (19 contacts), sex (22 contacts), race/ethnicity (case-patients of 96 contacts and 140 contacts), cough (case-patients of 14 contacts), hospitalization (case-patients of 4 contacts), outcome (case-patients of 33 contacts).

^e^Variables with *P* values <0.20 in the univariable analyses were included in the multivariate model. The multivariate model included case-patient age, case-patient cough, contact age, contact race/ethnicity, and whether the contact was the spouse of the case-patient.
