# Supplementary material for: Epidemiology of SARS-CoV-2 transmission and superspreading in Salt Lake County, Utah, March–May 2020
Source: PLoS One. 2023 Jun 23;18(6):e0275125. doi: 10.1371/journal.pone.0275125 (PMC10289415; doi:10.1371/journal.pone.0275125)
Supplement: S2 Table — (DOCX) [file pone.0275125.s003.docx]

**S2 Table. Secondary attack rates among 633 contacts in non-household settings based on case-patient^a^ characteristics, their own characteristics, and setting of transmission.**

|  | | | Univariate | | | Multivariate | |
| --- | --- | --- | --- | --- | --- | --- | --- |
| Case-patient characteristic |  | No. of Contacts | No. of Secondary Case-Patients (Secondary Attack Rate)^b^ | OR (95% CI) | *P* | OR (95% CI) | *P* |
| Age (years) | <18 | 2 | 0 (0%) | - | - |  |  |
|  | 18-44 | 388 | 64 (16%) | REFERENT |  |  |  |
|  | 45-64 | 221 | 34 (15%) | 0.92 (0.58–1.44) | 0.72 | 1.17 (0.68–1.98) | 0.58 |
|  | ≥65 | 22 | 6 (27%) | 1.90 (0.66–4.81) | 0.198^f^ | 3.53 (1.06–11.5) | 0.03 |
| Sex | Female | 342 | 49 (14%) | REFERENT |  |  |  |
|  | Male | 291 | 55 (19%) | 1.39 (0.91–2.13) | 0.12^f^ | 1.20 (0.73–1.97) | 0.48 |
| Race/Ethnicity^c,d^ | Non-Hispanic White | 284 | 47 (17%) | REFERENT |  |  |  |
|  | Hispanic or Non-White | 187 | 39 (21%) | 1.33 (0.83–2.13) | 0.24 |  |  |
| Cough^d^ | Yes | 510 | 79 (15%) | REFERENT |  |  |  |
|  | No | 110 | 23 (21%) | 1.44 (0.84–2.39) | 0.17^f^ | 1.80 (0.93–3.37) | 0.07 |
| Hospitalization^d^ | No | 565 | 92 (16%) | REFERENT |  |  |  |
|  | Yes | 67 | 12 (18%) | 1.12 (0.55–2.11) | 0.73 |  |  |
| Outcome^d^ | Died | 4 | 1 (25%) | 1.63 (0.08–12.9) | 0.67 |  |  |
|  | Recovered | 596 | 101 (17%) | REFERENT |  |  |  |
| Contact characteristics |  |  |  |  |  |  |  |
| Age (years)^d^ | <18 | 53 | 7 (13%) | 0.63 (0.25–1.37) | 0.28 |  |  |
|  | 18-44 | 359 | 70 (19%) | REFERENT |  |  |  |
|  | 45-64 | 147 | 23 (16%) | 0.77 (0.45–1.27) | 0.31 |  |  |
|  | ≥65 | 34 | 4 (12%) | 0.55 (0.16–1.45) | 0.28 |  |  |
| Sex^d^ | Female | 306 | 48 (16%) | REFERENT |  |  |  |
|  | Male | 392 | 56 (19%) | 1.22 (0.80–1.87) | 0.35 |  |  |
| Race/Ethnicity^d^ | Non-Hispanic White | 210 | 35 (17%) | REFERENT |  |  |  |
|  | Hispanic or Non-White | 208 | 59 (28%) | 1.98 (1.24–3.20) | <0.01^f^ | 1.75 (1.07–2.91) | 0.03 |
| Exposure setting^d^ |  |  |  |  |  |  |  |
| Type | Social | 212 | 48 (23%) | REFERENT |  |  |  |
|  | Work | 299 | 43 (14%) | 0.57 (0.36–0.90) | 0.02^f^ | 0.92 (0.54–1.54) | 0.74 |
|  | Healthcare | 38 | 2 (5%) | 0.19 (0.03–0.65) | 0.03^f^ | 0.46 (0.06–1.90) | 0.34 |
|  | Other^e^ | 45 | 6 (13%) | 0.53 (0.19–1.23) | 0.17^f^ | 0.68 (0.21–1.88) | 0.49 |

^a^In this table, case patients refer to laboratory confirmed case-patients identified in the community and reported to SLCoHD, as well as laboratory confirmed secondary case-patients identified through contact tracing who had their close contacts traced; probable (symptomatic but untested) secondary case-patients were excluded as case-patients from this analyses.

^b^Secondary attack rates among contacts were calculated as the proportion of contacts that were confirmed (tested positive SARS-CoV-2) or probable (symptomatic but untested) secondary case-patients.

^c^Hispanic or Non-White includes Hispanic; Black or African American, non-Hispanic; Asian, non-Hispanic; American Indian/Alaska Native, non-Hispanic; Native Hawaiian/Other Pacific Islander, non-Hispanic; or Two or More Races/Other, non-Hispanic.

^d^Data were missing for the following variables: age (40 contacts), sex (25 contacts), race/ethnicity (case-patients of 162 contacts and 215 contacts), cough (case-patients of 13 contacts), hospitalization (case-patients of 1 contact), outcome (case-patients of 33 contacts), exposure setting (39 contacts).

^e^Other non-household settings include school and daycare, conference, family members not living in the household, retail stores.

^f^Variables with *P* values <0.20 in the univariable analyses were included in the multivariate model. The multivariate model included case-patient age, case-patient sex, case-patient cough, contact race/ethnicity, and exposure setting.
