# Supplementary material for: Epidemiology of SARS-CoV-2 transmission and superspreading in Salt Lake County, Utah, March–May 2020
Source: PLoS One. 2023 Jun 23;18(6):e0275125. doi: 10.1371/journal.pone.0275125 (PMC10289415; doi:10.1371/journal.pone.0275125)
Supplement: S3 Table — (DOCX) [file pone.0275125.s004.docx]

**S3 Table. Number of secondary case-patients generated by 184 index case-patients based on case-patient characteristics.**

|  | | | Univariate | | | Multivariate | |
| --- | --- | --- | --- | --- | --- | --- | --- |
| Index  Case-patient characteristic |  | No. (%) of Index  Case-patients | % (n) of Index Case-Patients Generating 2+ Secondary  Case-Patients | OR (95% CI) | *P* | OR (95% CI) | *P* |
| Symptom  Onset Timing | Before  Stay-Home Directive | 92 (50%) | 35.9% (33) | REFERENT |  |  |  |
|  | During Directive | 92 (50%) | 45.7% (42) | 1.50 (0.83 – 2.73) | 0.18^c^ | 1.21 (0.63 - 2.31) | 0.57 |
| Age (years) | <18 | 3 (1.6%) | 0% (0) | <0.01 (0 - >100) | 0.99 | <0.01 (0 - >100) | 0.99 |
|  | 18-44 | 102 (55.4%) | 46.1% (47) | REFERENT |  |  |  |
|  | 45-64 | 61 (33.2%) | 39.3% (24) | 0.76 (0.40 – 1.44) | 0.40 | 0.80 (0.41 – 1.53) | 0.50 |
|  | ≥65 | 18 (9.8%) | 22.2% (4) | 0.33 (0.09 – 1.01) | 0.07^c^ | 0.36 (0.09 – 1.08) | 0.09 |
| Sex | Female | 93 (50.5%) | 40.9% (38) | REFERENT |  |  |  |
|  | Male | 91 (49.5%) | 40.7% (37) | 0.99 (0.55 – 1.79) | 0.98 |  |  |
| Race/Ethnicity^a,b^ | Non-Hispanic White | 86 (46.7%) | 34.9% (30) | REFERENT |  |  |  |
|  | Hispanic or Non-White | 82 (44.6%) | 48.8% (40) | 1.78 (0.96 – 3.32) | 0.07^c^ | 1.64 (0.84 – 3.20) | 0.14 |
| Cough^d^ | Yes | 133 (72.3%) | 42.1% (56) | REFERENT |  |  |  |
|  | No | 48 (26.1%) | 39.6% (19) | 0.90 (0.45 – 1.76) | 0.76 |  |  |
| Hospitalization^b^ | No | 164 (89.1%) | 40.2% (66) | REFERENT |  |  |  |
|  | Yes | 18 (9.8%) | 50% (9) | 1.48 (0.55 - 4.00) | 0.43 |  |  |
| Outcome^b^ | Died | 3 (1.6%) | 33.3% (1) | 0.74 (0.03 – 7.90) | 0.81 |  |  |
|  | Recovered | 179 (97.3%) | 40.2% (72) | REFERENT |  |  |  |
| Days from Symptom Onset to Isolation/Quarantine | | | | 1.04 (0.98 – 1.10) | 0.20 |  |  |

^a^Hispanic or Non-White includes Hispanic; Black or African American, non-Hispanic; Asian, non-Hispanic; American Indian/Alaska Native, non-Hispanic; Native Hawaiian/Other Pacific Islander, non-Hispanic; or Two or More Races/Other, non-Hispanic.

^b^Data were missing for the following variables: race/ethnicity (16 index case-patients), cough (3 index case-patients), hospitalization (2 index case-patients), outcome (2 index case-patients).

^c^Variables with *P* values <0.20 in the univariable analyses were included in the multivariate model. The multivariate model included symptom onset timing, age, and race/ethnicity.
